# Inferior Vena Caval Measures Do Not Correlate with Carotid Artery Corrected Flow Time Change Measured Using a Wireless Doppler Patch in Healthy Volunteers

**DOI:** 10.3390/diagnostics13233591

**Published:** 2023-12-03

**Authors:** Jon-Emile S. Kenny, Ross Prager, Philippe Rola, Garett McCulloch, Sarah Atwi, Chelsea E. Munding, Joseph K. Eibl, Korbin Haycock

**Affiliations:** 1 Health Sciences North Research Institute, Sudbury, ON P3E 2H3, Canada; 2 Flosonics Medical, Toronto, ON P3E 2H2, Canada; 3 Division of Critical Care Medicine, Western University, London, ON N6A 3K7, Canada; 4 Intensive Care Unit, Santa Cabrini Hospital, Montreal, QC H1T 1P7, Canada; 5 Northern Ontario School of Medicine, Sudbury, ON P3E 2C6, Canada; 6 Department of Emergency Medicine, Riverside University Health System Medical Center, Moreno Valley, CA 92555, USA

**Keywords:** inferior vena cava collapse, functional hemodynamic monitoring, common carotid artery corrected flow time, healthy volunteer

## Abstract

(1) Background: The inspiratory collapse of the inferior vena cava (IVC), a non-invasive surrogate for right atrial pressure, is often used to predict whether a patient will augment stroke volume (SV) in response to a preload challenge. There is a correlation between changing stroke volume (SV_∆_) and corrected flow time of the common carotid artery (ccFT_∆_). (2) Objective: We studied the relationship between IVC collapsibility and ccFT_∆_ in healthy volunteers during preload challenges. (3) Methods: A prospective, observational, pilot study in euvolemic, healthy volunteers with no cardiovascular history was undertaken in a local physiology lab. Using a tilt-table, we studied two degrees of preload augmentation from (a) supine to 30-degrees head-down and (b) fully-upright to 30-degrees head down. In the supine position, % of IVC collapse with respiration, sphericity index and portal vein pulsatility was calculated. The common carotid artery Doppler pulse was continuously captured using a wireless, wearable ultrasound system. (4) Results: Fourteen subjects were included. IVC % collapse with respiration ranged between 10% and 84% across all subjects. Preload responsiveness was defined as an increase in ccFT_∆_ of at least 7 milliseconds. A total of 79% (supine baseline) and 100% (head-up baseline) of subjects were preload-responsive. No supine venous measures (including IVC % collapse) were significantly related to ccFT_∆_. (5) Conclusions: From head-up baseline, 100% of healthy subjects were ‘preload-responsive’ as per the ccFT_∆_. Based on the 42% and 25% IVC collapse thresholds in the supine position, only 50% and 71% would have been labeled ‘preload-responsive’.

## 1. Introduction

Detecting preload (or fluid) responsiveness is of emerging interest, especially in the intensive care unit (ICU). The basis of preload responsiveness is using the change in volume or flow—that is, stroke volume or cardiac output—from the heart to determine if intravenous (IV) fluids are effective, rather than other commonly used clinical measures (e.g., blood pressure, urine output) [1,2,3]. Decades of research has demonstrated that traditional bedside measures are notoriously poor at determining cardiac output change [4,5]. For example, when there is a clinically significant increase in cardiac output, less than 50% of patients experience an increase in mean arterial pressure [6,7]; in other words, the sensitivity of blood pressure is particularly poor for gauging the effect of IV fluids in sepsis.

Though the clinical benefit of using preload responsiveness (i.e., changing stroke volume or cardiac output) to guide care is controversial [8], association studies [9] and one large randomized controlled trial [10] demonstrated that administering IV fluid only when a patient is both hypo-perfused (e.g., hypotensive, skin mottling, organ dysfunction) and preload-responsive improved outcome. This approach is generally tied to administering less IV fluid than usual care, which saves patients the downstream complications of excessive fluid volume such as mechanical ventilation and dialysis [11]. These results are in line with earlier data in patients with acute respiratory distress syndrome (i.e., ARDS), many of whom had septic sources [12,13]. Various ultrasonographic methods are used in the ICU to detect preload responsiveness, including respiratory variation in the inferior vena cava [14] and carotid Doppler ultrasound [15,16]. Nevertheless, little is known about the characteristics of these measures in the normal, healthy state. 

In spontaneously breathing subjects (i.e., those breathing without controlled mechanical ventilation), the inspiratory collapse of the inferior vena cava was initially described as a non-invasive surrogate for right atrial pressure [17,18]. Indeed, this relationship is well-accepted when calculating right ventricular systolic pressure via echocardiography [19]. Yet, there is a poor relationship between right atrial pressure and the cardiac response to preload (i.e., ‘volume’ or ‘preload responsiveness’) [20], which may explain conflicting data for inspiratory IVC collapse as a surrogate for preload responsiveness [14].

Like the inspiratory collapse of the IVC, the corrected flow time of the common carotid artery (ccFT) has been studied as a surrogate to predict stroke volume change (SV_∆_) in the critically ill [21] and elective surgical populations [15,22]. The duration of systole (i.e., flow time in milliseconds) is determined largely by heart rate, afterload, contractility and SV [23]. Applying a mathematical correction for heart rate (e.g., Wodey equation [23]) yields the corrected flow time which better approximates SV and especially its change. To investigate whether ccFT measured using the wearable ultrasound is a surrogate for SV_∆_—and, therefore, for preload responsiveness—we have shown in healthy volunteers that there is a strong, linear relationship between changing ccFT (ccFT_∆_) and SV_∆_ using non-invasive pulse contour analysis, bioreactance and aortic Doppler velocity as gold standards [23,24,25,26]. Furthermore, in these studies comprising roughly 90 preload augmentations in approximately 50 healthy subjects, SV_∆_ of at least +10% (i.e., preload responsiveness) was ubiquitous and the optimal ccFT_∆_ threshold for detecting +10% SV_∆_ was above +2 ccFT_∆_. We have recently shown that a 10% SV_∆_ measured using ascending aortic Doppler correlates with an absolute +7 ms ccFT_∆,_ identical to the +7 ms value ascertained by Barjaktarevic and colleagues using a hand-held Doppler of the carotid artery in patients with undifferentiated shock [21]. Lastly, in elective coronary artery bypass grafted patients, preliminary data from the wearable Doppler echo these findings [27].

In addition to IVC collapse and ccFT, additional ultrasound measures are used to guide resuscitation in the intensive care unit. For instance, the venous excess ultrasound score (VExUS) was recently described [28], which comprises measurement of IVC size and venous Doppler morphology of the portal, hepatic and intra-renal veins [29]. In pathological states such as volume overload, the VExUS score is related to acute kidney injury [30] and was recently shown to be associated with right atrial pressure [31]. Though high VExUS is helpful in identifying an abnormal relationship between venous return and cardiac function, its ability to predict the hemodynamic state of healthy volunteers is limited.

To our knowledge, no study has related inspiratory IVC collapse or components of the VExUS score to ccFT_∆_ during preload augmentation in healthy volunteers. In this pilot investigation, we evaluated three hypotheses. First, we expected that, in this cohort of healthy volunteers, preload augmentation via the Trendelenburg position would increase ccFT by a clinically significant degree (i.e., ≥7 ms or +2% ccFT_∆_) in the vast majority of subjects. In other words, the fraction of ‘preload-responsive’ subjects would be congruent with earlier findings in healthy volunteers [23,24,26,32]. Second, we predicted that preload responsiveness would be accompanied by a collapsing IVC (i.e., >25%), based on the previous literature [33]. Finally, we explored whether other venous measures, such as the end-expiratory IVC diameter, sphericity index [34] and portal vein pulsatility [28], correlated with ccFT_∆_ during preload augmentation.

## 2. Materials and Methods

### 2.1. Clinical Setting

The study was reviewed and approved by the research ethics board of Health Sciences North (#19-011). A convenience sample of healthy subjects was recruited at a local physiology lab. Written informed consent was obtained from all subjects. Inclusion criteria were healthy, adult, clinically euvolemic volunteers able to give informed consent. Exclusion criteria were known cardiovascular history and/or taking regular cardiovascular medications. 

### 2.2. Carotid Artery Doppler Measures

**Figure 1:**
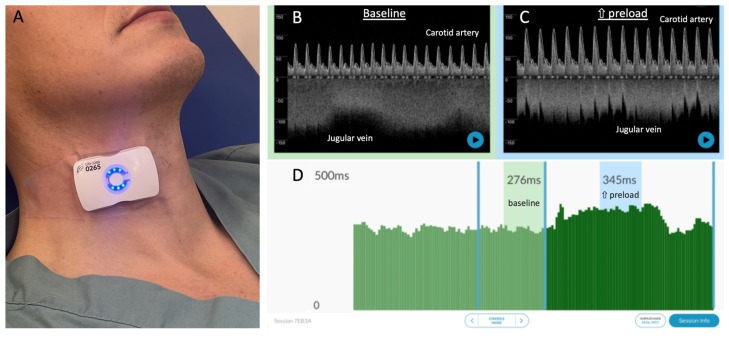
The wearable Doppler system and its graphical user interface. (**A**) The wireless, wearable Doppler transducer adhered to the neck of a healthy subject. (**B**) Representative resting baseline data from a healthy subject with common carotid and internal jugular spectrograms. (**C**) Representative data during increased preload showing carotid and jugular spectrograms; note the change in carotid and jugular morphologies consistent with increased preload and ccFT. (**D**) Per-beat ccFT calculations showing windows at baseline (average ccFT 276 ms) and during increased preload (average ccFT 345 ms).

A carotid artery Doppler was obtained using a novel, wireless, wearable ultrasound system (Flosonics Medical, Sudbury, ON, Canada) that is FDA-cleared. The device is a 4 MHz, continuous-wave Doppler ultrasound that generates a 2 cm wide and 4 cm deep sonic curtain and is placed without image guidance. As previously described [23,24,26], the common carotid artery Doppler spectrogram is obtained via simultaneously acquired visual and audio cues from the wearable system. Given that a normal common carotid artery is 6–7 mm in diameter, the 2 cm ultrasound beam generated by the wearable device generally insonates all red blood cells moving through the artery. The wearable system was placed on the neck while the subject was in the head-up position on the tilt-table by an expert with the device (JESK, JKE). Once an identifiable signal was seen and heard, the wearable Doppler patch was adhered in place on the neck; the continuous carotid Doppler was monitored throughout the entire protocol (Figure 1). The flow time of the carotid artery was calculated as the time from systolic upstroke to the dicrotic notch. The flow time was then corrected for heart rate based on the equation of Wodey, as described by Barjaktarevic and colleagues [21]:

$$corrected\;flow\;time=flow\;time+\left[1.29\times \left(heart\;rate-60\right)\right].$$

We have previously shown that, in healthy volunteers, there was no significant difference in the various corrected flow time equations for detecting stroke volume change [23].

### 2.3. Venous Measures

The percent collapse of the inferior vena cava, sphericity index and portal vein pulsatility were obtained by an expert sonographer (R.P., P.R., K.H.), as previously described [28,34]. Ultrasound studies were performed using a Sparq system (Philips Healthcare, Amsterdam, The Netherlands) with a phased array or a convex array transducer. Subjects were positioned in a dorsal decubitus position during the examination with the tilt-table at 0°. Measurements were made at end-expiration. Intrarenal Doppler assessment was performed by pulsed wave Doppler waveform capture at the corticomedullary junction during a respiratory pause after the end of expiration to obtain 2 to 3 consecutive cardiac cycles.

All venous calculations are listed below:

$$IVC\;collapse\;\%=\frac{\left({diameter}_{end-exp}-{diameter}_{end-insp}\right)}{{diameter}_{end-exp}}$$

$$IVC\;sphericity\;index=\frac{short\;diameter}{long\;diameter}$$

$$Portal\;vein\;pulsatility\;index=\frac{maximum\;velocity-minimum\;velocity}{max\;velocity}.$$

### 2.4. Tilt-Table Protocol

The protocol for this investigation consisted of 3 gravitational positions on a tilt-table: supine, fully upright (i.e., head up) and head-down tilt (30 degrees below the horizontal line). Each protocol began in the supine position and each subject had an ultrasound examination performed by an expert in the field, as described above. That is, the venous measures including IVC collapse were recorded in the resting, supine position. Once baseline venous measures were completed by the sonographer, the subject was tilted upwards into the fully-upright position for repeat measures and then downwards to the head-down position, as described previously [35]. Via the wearable ultrasound system, common carotid artery Doppler measures were recorded throughout the entire protocol and stored for offline analysis.

### 2.5. Analysis

The analysis was then completed in three parts. First, for each subject, the ccFT_∆_ was calculated from the fully upright as well as supine to head-down position. The ccFT_∆_ was evaluated based on its maximal change for each level of preload. The number of cardiac cycles averaged in the baseline (i.e., head-up and supine) and head-down positions was based upon a section of recording with the lowest ccFT coefficient of variation; this was carried out to ensure a stable signal in the ccFT and to obtain an adequate number of beats to detect maximal change with statistical confidence. Any regions with obvious artifacts (e.g., vocalization, deglutition) were omitted. Second, inspiratory IVC collapse at baseline was calculated and plotted against ccFT_∆_ for each subject; linear regression was performed to assess for correlation. Prior to performing linear regressions, we tested for the assumption of normality of each variable using the Shapiro–Wilk test (Python V3.9.12, Scipy V1.7.3). Finally, linear regression was performed to assess for an association between end-expiratory IVC diameter, sphericity index and portal vein pulsatility and the % change and absolute ccFT_∆_. Pearson correlation coefficients for all combinations of venous measures versus maximum ccFT_∆_ were calculated.

## 3. Results

### 3.1. Patients

Fifteen adult volunteers were studied; eight were female. One male subject was entirely excluded because of lost ultrasound image uploads; therefore, the analysis comprised fourteen adults in total. The baseline characteristics of the healthy volunteers included in the final analysis are listed in Table 1.

### 3.2. Supine Venous Measures

The mean inspiratory IVC collapse for all subjects at supine baseline was 44% and the range of values was between 9.7% and 83.1%. These values for end-expiratory IVC diameter, sphericity index and portal vein pulsatility were 1.18 cm (0.42 cm to 2.36 cm), 0.56 (0.37 to 0.98) and 24.4% (13.3% to 37.4%), respectively.

### 3.3. Head-Up Baseline ccFT_∆_

From head-up to head-down, the average, maximal percent (%) and absolute ccFT_∆_ were 19.3% and 54.4 milliseconds (ms), respectively. For all subjects, the ranges for maximal % and absolute ccFT_∆_ were 10.9% to 28.7% and 32.8 to 81.3 ms, respectively. With preload responsiveness defined as an increase in ccFT_∆_ of at least 7 milliseconds, 100% of the subjects met this threshold from head-up baseline.

### 3.4. Supine Baseline ccFT_∆_

From supine to head-down, the average maximal percent (%) and average absolute ccFT_∆_ were 4.4% and 14.0 milliseconds (ms), respectively. For all subjects, the ranges for maximal % and absolute ccFT_∆_ were 0% to 12.8% and 0.1 to 38.9 ms, respectively. With preload responsiveness defined as an increase in ccFT_∆_ of at least 7 milliseconds, 79% of the subjects met this threshold from supine baseline.

### 3.5. Relationship between Supine Venous Measures and ccFT_∆_

Each variable, except for the supine sphericity index compared to supine baseline ccFT, revealed a non-significant Shapiro–Wilk *p*-value (*p* > 0.05), indicating that data were drawn from a normal distribution. We log-transformed the supine sphericity index during the supine baseline, which passed normality testing (*p* > 0.05), and we used it in subsequent linear regression.

**Figure 2:**
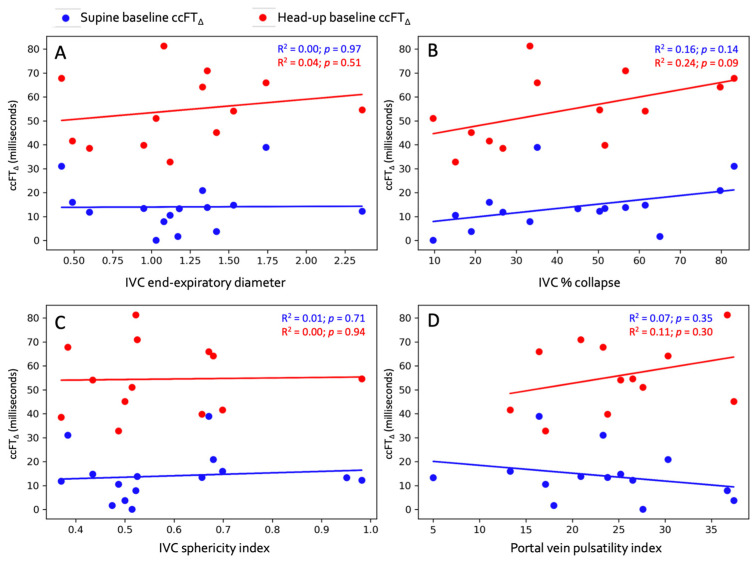
Linear regression between supine venous measures and absolute ccFT_∆_. (**A**) End-expiratory IVC diameter. (**B**) IVC collapse %. (**C**) IVC sphericity index. (**D**) Portal vein pulsatility index. Blue dots represent supine baseline and red dots represent head-up baseline.

The regression between supine venous measures and maximal absolute ccFT_∆_ is shown in Figure 2. The Pearson correlation coefficients for all venous measures versus both absolute and % ccFT_∆_ are shown in Table 2.

## 4. Discussion

In this pilot study evaluating the relationship between supine ultrasonographic venous measures (e.g., inspiratory IVC collapse) and ccFT_∆_ during two degrees of preload augmentation, we report several clinically relevant findings. First, we found that the degree of preload augmentation affected the proportion of healthy subjects who were considered preload-responsive. Second, all healthy volunteers were ultimately ‘preload-responsive’ based on ccFT_∆_ with a large enough increase in venous return. Third, the range of baseline, supine, inspiratory IVC collapse values in this euvolemic populationwas broad (i.e., between 10% and 85% collapse). Finally, there was no statistical relationship between baseline venous measures and ccFT_∆_ during either degree of preload augmentation.

The ‘dose response’ to preload augmentation and its impact on the number of participants labeled preload-responsive by ccFT_∆,_ is clinically important. Trendelenburg positioning is reported to accurately predict patients who respond to intravenous fluid administration; however, the maneuver itself is variably practiced. For example, Ma and colleagues moved patients from 15 degrees head above the horizontal to 15 degrees below; this resulted in an excellent diagnostic accuracy with 100% sensitivity [36]. On the other hand, Terai and colleagues performed the maneuver by comparing the horizontal (i.e., supine) to head-down in healthy subjects [37]. They found a significant rise in SV within the first minute of head down, though individual SV% increase was not reported so the fraction of ‘preload-responsive’ subjects could not be calculated. These findings are congruent with the passive leg raise (PLR) literature where the greatest hemodynamic effect occurs when lowering the heart from head-up position; raising the legs above the heart has less impact [38]. Our findings also explain data from Godfrey and colleagues [39] who performed a modified PLR in healthy volunteers and found that only 50% of subjects increased their SV by at least 10%. In their study, the baseline was the fully supine position and the preload challenge consisted of only raising the legs. Based on our observations, it is quite likely that the modified PLR by Godfrey et al. did not recruit enough venous blood to trigger the Starling mechanism [40]. Importantly, in previous work, we have shown that determining ‘adequate’ preload augmentation might be assessed via the jugular Doppler spectrum obtained using the wearable ultrasound system [35]. Only the change in the jugular Doppler morphology accurately tracked the preload state in comparison to IVC collapse % and Doppler of the hepatic, portal and intra-renal veins [35]. From the fully upright to head-down position, 13/14 healthy subjects changed from a continuous to pulsatile jugular waveform, whereas only 1/14 showed this change from supine to head-down. Therefore, confirming the change in jugular venous morphology helps identify adequate venous return; this could improve the sensitivity of detecting preload responsiveness. 

Second, the fact that all healthy volunteers were ultimately preload-responsive with a large enough increase in venous return is congruent with previous data in this population [23,24,32]. For instance, in healthy volunteers raising preload via the squat maneuver, a clinically significant SV rise was always observed [23]. Additionally, all healthy volunteers increased SV with the release of lower body negative pressure (LBNP). Including this current study, data from over 60 healthy volunteers undergoing approximately 100 preload augmentations are reported, with all subjects having a clinically significant rise in ccFT. From this, we surmise that ‘preload responsiveness’ is a normal physiologic state in euvolemic, ambulatory, healthy volunteers. The relationship between ccFT_∆_ and SV_∆_ is clinically important as non-invasively inferring SV_∆_ at the bedside can help guide resuscitation. For example, if preload is administered and there is little ccFT_∆_, the patient may be judged as fluid ‘unresponsive’ and another intervention may be attempted. Another potential implication is detecting diminishing SV (e.g., hemorrhage), especially when mean arterial pressure is maintained by increased vascular resistance [24].

Third, contrary to what we anticipated, there was a wide range of inspiratory IVC collapse at the baseline supine position in these healthy, clinically euvolemic volunteers; that is, IVC collapse was between 10% and 85%. Given that Corl and colleagues previously observed that an IVC collapse of 25% best dichotomized fluid responders and non-responders [33], we suspected that all of our healthy subjects would have displayed an IVC collapsibility greater than 25%. Contrary to this, approximately 29% of the subjects in our study had an IVC collapse of less than 25%, meaning that almost one-third of these healthy subjects would have been labeled as fluid ‘unresponsive’—a value observed in the early septic shock population [41,42]. Moreover, Airapetian et al. identified 42% IVC collapse as the best threshold for identifying fluid responsive patients; based on this value, 50% of these healthy subjects would have been labeled as ‘preload-unresponsive’. Importantly, we found no statistically significant correlation between IVC collapse and ccFT_∆_ during both degrees of preload augmentation, implying that the respiratory variation in the IVC did not predict SV_∆_ in this paradigm. This small, pilot study cannot explain the underlying mechanism of this observation, though the inspiratory collapse of the IVC is driven by multiple determinants, only one of which is the pressure within the vein. Subtle differences in blood volume, inspiratory pattern, abdominal compliance and the distribution between portal and non-portal venous return may mediate the size and collapse of the IVC [18,43,44,45,46,47,48,49,50,51,52]. The dissociation between the IVC collapse and ccFT_∆_ is clinically relevant, as measuring ccFT_∆_ ostensibly improves the sensitivity of IVC collapse for predicting fluid responders. In other words, ccFT_∆_ in this study identified the 29% and 50% of healthy subjects who would have been falsely labeled as fluid-unresponsive (i.e., false negatives) based on the 25% and 42% IVC collapse thresholds, respectively.

Finally, as a hypothesis-generating analysis, we explored the relationship between other venous measures and ccFT_∆_ at different levels of preload augmentation. Given that the IVC is a three-dimensional structure, we were particularly curious as to whether the sphericity index—which is a good approximation of right atrial pressure [34]—would be associated with ccFT_∆_. The IVC sphericity index is measured at end-expiration and is the ratio of the IVC short-to-long diameter in the transverse plane. Based on the Starling curve [53], venous measures intimating low right atrial pressure (i.e., small end-expiratory IVC diameter, small sphericity index and low portal pulsatility) should associate with large ccFT_∆_. No clear relationship was observed between any supine venous measure with ccFT_∆_ from either supine or head-up baseline. At least in healthy volunteers, these data imply that these venous measures may not accurately predict preload responsiveness in this group. These discrepant results may ultimately be reconciled if both venous and arterial measures are tracked throughout a preload challenge. Traditionally, this entails measuring stroke volume and filling pressure (e.g., central venous pressure) before, during and after a preload challenge. With this data, the concurrently plotted change in venous pressure and arterial output gives the slope of the cardiac function curve. Though this is challenging with ultrasound, the simultaneous Doppler of a major artery and vein could be a window to the right and left heart synchronously [54,55].

Our study has several important limitations. We did not measure the SV_∆_ during head-down positioning, thus we cannot know for certain the functional cardiac state of the healthy subjects we studied. Nevertheless, Trendelenburg is a well-accepted means of increasing preload and testing functional hemodynamics [36,37,56]. In addition, we found a strong relationship between SV_∆_ and ccFT_∆_ in this population; a +2% ccFT_∆_ threshold is an excellent surrogate for +10% SV_∆_ [23,24,26]. Therefore, we have confidence that all subjects were ‘preload-responsive’ despite not directly measuring SV_∆_. All subjects also increased their ccFT well above the optimal threshold for detecting +10% SV_∆_, as observed by Barjaktarevic and colleagues in undifferentiated shock [21]. Though not all investigators have found ccFT to reliably detect SV_∆_ [57], we note that many of these studies suffer from human measurement variability [58], limited cardiac cycle sample size (i.e., sampling only a few beats before and during an intervention) and confounders introduced by gold-standard algorithm lag [23,26]. The wearable Doppler system allowed us to minimize measurement variability as it is adhered in place on the neck and thousands of cardiac cycles were sampled across the entire preload challenge for all subjects. Second, this study was conducted in healthy volunteers, limiting our ability to apply these results to patients with hemodynamic derangement. Other investigators have found inspiratory IVC collapse to have a better diagnostic accuracy when patients performed standardized inspiratory maneuvers [59]; doing so may have improved the relationship between IVC collapse and ccFT_∆_ in our study. Third, the accuracy of IVC collapse is affected by sonographer experience [60]; however, the three sonographers who performed B-mode imaging of the IVC and portal Doppler measurements are recognized experts in this field with decades of combined experience, so we believe user error is less likely to have been a problem in our investigation.

## 5. Conclusions

All healthy volunteers in this study had a clinically significant rise in ccFT when moved from upright to head-down position, strongly suggesting that all subjects had a +10% stroke volume augmentation; a total of 79% had this response when moved from supine to head-down. Therefore, the degree of preload augmentation is an important determinant when assessing preload responsiveness. The inspiratory collapse of the IVC in the supine position was poorly related to the change in ccFT. Based on the 42% and 25% IVC collapse thresholds, 50% and 29% of these healthy subjects would have been labeled ‘preload-unresponsive’, which is comparable to the critically ill population and not to healthy subjects. Finally, other venous measures in the supine position, including IVC sphericity index and portal vein Doppler pulsatility, did not strongly correlate with ccFT_∆_. Further investigation in patients with hemodynamic pathology is needed to confirm these preliminary observations.

## Figures and Tables

**Table 1:** Baseline characteristics and measures in the supine position. m: meters; kg/m^2^: kilograms per meters-squared; mmHg: millimeters of mercury; bpm: beats per minute; ccFT: corrected flow time of the carotid artery.

*n* = 14	Mean	Std
Patient Age	29.6	±9.3
Patient Height (m)	1.7	±0.1
Patient Weight (kg)	69.1	±16.0
BMI (kg/m^2^)	23.1	±4.2
MAP (mmHg)	96.3	±10.6
HR (bpm)	73.5	±10.6
Systolic Blood Pressure (mmHg)	127.7	±16.3

**Table 2:** Regression results.

Corrected Carotid Flow Time_∆_		Supine Venous Measures			
		IVC_EE_	IVC_%_	SI_IVC_	PI_PORTAL_
Head-up baseline	ccFT absolute_∆_	R^2^ = 0.041	R^2^ = 0.25	R^2^ = 0.0006	R^2^ = 0.11
	ccFT %_∆_	R^2^ = 0.029	R^2^ = 0.29	R^2^ = 0.0009	R^2^ = 0.097
Supine baseline	ccFT absolute_∆_	R^2^ = 0.0001	R^2^ = 0.16	R^2^ = 0.011	R^2^ = 0.075
	ccFT %_∆_	R^2^ = 0.000008	R^2^ = 0.16	R^2^ = 0.0095	R^2^ = 0.077

## Data Availability

Data available upon reasonable request.

## References

[1] Bentzer P., Griesdale D.E., Boyd J., MacLean K., Sirounis D., Ayas N.T. (2016). Will this hemodynamically unstable patient respond to a bolus of intravenous fluids?. JAMA.

[2] Michard F., Teboul J.-L. (2002). Predicting fluid responsiveness in ICU patients: A critical analysis of the evidence. Chest.

[3] Michard F., Boussat S., Chemla D., Anguel N., Mercat A., Lecarpentier Y., Richard C., Pinsky M.R., Teboul J.-l. (2000). Relation between respiratory changes in arterial pulse pressure and fluid responsiveness in septic patients with acute circulatory failure. Am. J. Respir. Crit. Care Med..

[4] Marik P., Bellomo R. (2015). A rational approach to fluid therapy in sepsis. BJA Br. J. Anaesth..

[5] Marik P.E. (2016). Fluid responsiveness and the six guiding principles of fluid resuscitation. Crit. Care Med..

[6] García M.I.M., González P.G., Romero M.G., Cano A.G., Oscier C., Rhodes A., Grounds R.M., Cecconi M. (2015). Effects of fluid administration on arterial load in septic shock patients. Intensive Care Med..

[7] Magder S., Bafaqeeh F. (2007). The clinical role of central venous pressure measurements. J. Intensive Care Med..

[8] Ehrman R.R., Gallien J.Z., Smith R.K., Akers K.G., Malik A.N., Harrison N.E., Welch R.D., Levy P.D., Sherwin R.L. (2019). Resuscitation Guided by Volume Responsiveness Does Not Reduce Mortality in Sepsis: A Meta-Analysis. Crit. Care Explor..

[9] Dubin A., Loudet C., Kanoore Edul V.S., Osatnik J., Ríos F., Vásquez D., Pozo M., Lattanzio B., Pálizas F., Klein F. (2020). Characteristics of resuscitation, and association between use of dynamic tests of fluid responsiveness and outcomes in septic patients: Results of a multicenter prospective cohort study in Argentina. Ann. Intensive Care.

[10] Douglas I.S., Alapat P.M., Corl K.A., Exline M.C., Forni L.G., Holder A.L., Kaufman D.A., Khan A., Levy M.M., Martin G.S. (2020). Fluid response evaluation in sepsis hypotension and shock: A randomized clinical trial. Chest.

[11] Satterwhite L., Latham H. (2020). Fluid Management in Sepsis Hypotension and Septic Shock: Time to Transition the Conversation From Fluid Responsive to Fluid Refractory?. Chest.

[12] Wiedemann H., Wheeler A., Bernard G., Thompson B. (2006). Comparison of Two Fluid-Management Strategies in Acute Lung Injury. N. Engl. J. Med..

[13] Liu K.D., Thompson B.T., Ancukiewicz M., Steingrub J.S., Douglas I.S., Matthay M.A., Wright P., Peterson M.W., Rock P., Hyzy R.C. (2011). Acute kidney injury in patients with acute lung injury: Impact of fluid accumulation on classification of acute kidney injury and associated outcomes. Crit. Care Med..

[14] Orso D., Paoli I., Piani T., Cilenti F.L., Cristiani L., Guglielmo N. (2020). Accuracy of Ultrasonographic Measurements of Inferior Vena Cava to Determine Fluid Responsiveness: A Systematic Review and Meta-Analysis. J. Intensive Care Med..

[15] Beier L., Davis J., Esener D., Grant C., Fields J.M. (2020). Carotid Ultrasound to Predict Fluid Responsiveness: A Systematic Review. J. Ultrasound Med..

[16] Singla D., Gupta B., Varshney P., Mangla M., Walikar B.N., Jamir T. (2023). Role of carotid corrected flow time and peak velocity variation in predicting fluid responsiveness: A systematic review and meta-analysis. Korean J. Anesthesiol..

[17] Kircher B.J., Himelman R.B., Schiller N.B. (1990). Noninvasive estimation of right atrial pressure from the inspiratory collapse of the inferior vena cava. Am. J. Cardiol..

[18] Bodson L., Vieillard-Baron A. (2012). Respiratory variation in inferior vena cava diameter: Surrogate of central venous pressure or parameter of fluid responsiveness? Let the physiology reply. Crit. Care.

[19] Amsallem M., Sternbach J.M., Adigopula S., Kobayashi Y., Vu T.A., Zamanian R., Liang D., Dhillon G., Schnittger I., McConnell M.V. (2016). Addressing the Controversy of Estimating Pulmonary Arterial Pressure by Echocardiography. J. Am. Soc. Echocardiogr..

[20] Eskesen T., Wetterslev M., Perner A. (2016). Systematic review including re-analyses of 1148 individual data sets of central venous pressure as a predictor of fluid responsiveness. Intensive Care Med..

[21] Barjaktarevic I., Toppen W.E., Hu S., Montoya E.A., Ong S., Buhr R., David I.J., Wang T., Rezayat T., Chang S.Y. (2018). Ultrasound Assessment of the Change in Carotid Corrected Flow Time in Fluid Responsiveness in Undifferentiated Shock. Crit. Care Med..

[22] Jung S., Kim J., Na S., Nam W.S., Kim D.-H. (2021). Ability of Carotid Corrected Flow Time to Predict Fluid Responsiveness in Patients Mechanically Ventilated Using Low Tidal Volume after Surgery. J. Clin. Med..

[23] Kenny J.-É.S., Barjaktarevic I., Mackenzie D.C., Eibl A.M., Parrotta M., Long B.F., Eibl J.K. (2020). Diagnostic characteristics of 11 formulae for calculating corrected flow time as measured by a wearable Doppler patch. Intensive Care Med. Exp..

[24] Kenny J.-É.S., Barjaktarevic I., Mackenzie D.C., Elfarnawany M., Yang Z., Eibl A.M., Eibl J.K., Kim C.-H., Johnson B.D. (2021). Carotid Doppler ultrasonography correlates with stroke volume in a human model of hypovolaemia and resuscitation: Analysis of 48 570 cardiac cycles. Br. J. Anaesth..

[25] Kerrebijn I., Atwi S., Horner C., Elfarnawany M., Eibl A.M., Eibl J.K., Taylor J.L., Kim C.H., Johnson B.D., Kenny J.S. (2023). Correlation between changing carotid artery corrected flow time and ascending aortic Doppler flow velocity. Br. J. Anaesth..

[26] Kenny J.S., Barjaktarevic I., Eibl A.M., Parrotta M., Long B.F., Elfarnawany M., Eibl J.K. (2022). Temporal concordance between pulse contour analysis, bioreactance and carotid doppler during rapid preload changes. PLoS ONE.

[27] Kenny J.S., Clarke G., Kerrebijn I., Savery T., Knott M., Munding C.E., Elfarnawany M., Eibl A.E., Eibl J.K., Nalla B. (2023). Carotid Artery Corrected Flow Time Detects Stroke Volume Change Measured by Trans-Esophageal Echocardiography. Intensive Care Med. Exp..

[28] Beaubien-Souligny W., Rola P., Haycock K., Bouchard J., Lamarche Y., Spiegel R., Denault A.Y. (2020). Quantifying systemic congestion with Point-Of-Care ultrasound: Development of the venous excess ultrasound grading system. Ultrasound J..

[29] Argaiz E.R. (2021). VExUS Nexus: Bedside Assessment of Venous Congestion. Adv. Chronic. Kidney Dis..

[30] Spiegel R., Teeter W., Sullivan S., Tupchong K., Mohammed N., Sutherland M., Leibner E., Rola P., Galvagno S.M., Murthi S.B. (2020). The use of venous Doppler to predict adverse kidney events in a general ICU cohort. Crit. Care.

[31] Longino A., Martin K., Leyba K., Siegel G., Gill E., Douglas I.S., Burke J. (2023). Correlation between the VExUS score and right atrial pressure: A pilot prospective observational study. Crit. Care.

[32] Søndergaard S. (2022). Observational study on passive leg raising and the autonomic nervous system. Physiol. Rep..

[33] Corl K.A., George N.R., Romanoff J., Levinson A.T., Chheng D.B., Merchant R.C., Levy M.M., Napoli A.M. (2017). Inferior vena cava collapsibility detects fluid responsiveness among spontaneously breathing critically-ill patients. J. Crit. Care.

[34] Seo Y., Iida N., Yamamoto M., Machino-Ohtsuka T., Ishizu T., Aonuma K. (2017). Estimation of Central Venous Pressure Using the Ratio of Short to Long Diameter from Cross-Sectional Images of the Inferior Vena Cava. J. Am. Soc. Echocardiogr..

[35] Kenny J.-E.S., Prager R., Rola P., McCulloch G., Eibl J.K., Haycock K. (2023). The effect of gravity-induced preload change on the venous excess ultrasound (VExUS) score and internal jugular vein Doppler in healthy volunteers. Intensive Care Med. Exp..

[36] Ma G.-G., Xu L.-Y., Luo J.-C., Hou J.-Y., Hao G.-W., Su Y., Liu K., Yu S.-J., Tu G.-W., Luo Z. (2021). Change in left ventricular velocity time integral during Trendelenburg maneuver predicts fluid responsiveness in cardiac surgical patients in the operating room. Quant. Imaging Med. Surg..

[37] Terai C., Anada H., Matsushima S., Shimizu S., Okada Y. (1995). Effects of mild Trendelenburg on central hemodynamics and internal jugular vein velocity, cross-sectional area, and flow. Am. J. Emerg. Med..

[38] Jabot J., Teboul J.L., Richard C., Monnet X. (2009). Passive leg raising for predicting fluid responsiveness: Importance of the postural change. Intensive Care Med..

[39] Godfrey G.E., Dubrey S.W., Handy J.M. (2014). A prospective observational study of stroke volume responsiveness to a passive leg raise manoeuvre in healthy non-starved volunteers as assessed by transthoracic echocardiography. Anaesthesia.

[40] Monnet X., Teboul J.-L. (2008). Passive leg raising. Intensive Care Med..

[41] Hernández G., Ospina-Tascón G.A., Damiani L.P., Estenssoro E., Dubin A., Hurtado J., Friedman G., Castro R., Alegría L., Teboul J.-L. (2019). Effect of a resuscitation strategy targeting peripheral perfusion status vs serum lactate levels on 28-day mortality among patients with septic shock: The ANDROMEDA-SHOCK randomized clinical trial. JAMA.

[42] Kattan E., Ospina-Tascón G.A., Teboul J.-L., Castro R., Cecconi M., Ferri G., Bakker J., Hernández G., Investigators A.-S. (2020). Systematic assessment of fluid responsiveness during early septic shock resuscitation: Secondary analysis of the ANDROMEDA-SHOCK trial. Crit. Care.

[43] Millington S.J. (2019). Ultrasound assessment of the inferior vena cava for fluid responsiveness: Easy, fun, but unlikely to be helpful. Can. J. Anesth./J. Can. D’anesthésie.

[44] Millington S.J., Koenig S. (2021). Ultrasound Assessment of the Inferior Vena Cava for Fluid Responsiveness: Making the Case for Skepticism. J. Intensive Care Med..

[45] Via G., Tavazzi G., Price S. (2016). Ten situations where inferior vena cava ultrasound may fail to accurately predict fluid responsiveness: A physiologically based point of view. Intensive Care Med..

[46] Juhl-Olsen P., Frederiksen C.A., Hermansen J.F., Jakobsen C.J., Sloth E. (2012). Echocardiographic Measures of Diastolic Function Are Preload Dependent during Triggered Positive Pressure Ventilation: A Controlled Crossover Study in Healthy Subjects. Crit. Care Res. Pract..

[47] Juhl-Olsen P., Vistisen S.T., Christiansen L.K., Rasmussen L.A., Frederiksen C.A., Sloth E. (2013). Ultrasound of the inferior vena cava does not predict hemodynamic response to early hemorrhage. J. Emerg. Med..

[48] Juhl-Olsen P., Frederiksen C.A., Sloth E. (2012). Ultrasound assessment of inferior vena cava collapsibility is not a valid measure of preload changes during triggered positive pressure ventilation: A controlled cross-over study. Ultraschall. Med..

[49] Koratala A. (2023). Isolated Inferior Vena Cava Ultrasound in Hyponatremia: The Power and Peril of Point-of-Care Imaging. J. Ultrasound Med..

[50] Rola P., Haycock K., Spiegel R. (2023). What every intensivist should know about the IVC. J. Crit. Care.

[51] Di Nicolò P., Tavazzi G., Nannoni L., Corradi F. (2023). Inferior Vena Cava Ultrasonography for Volume Status Evaluation: An Intriguing Promise Never Fulfilled. J. Clin. Med..

[52] Kory P. (2017). COUNTERPOINT: Should Acute Fluid Resuscitation Be Guided Primarily by Inferior Vena Cava Ultrasound for Patients in Shock? No. Chest.

[53] Berlin D.A., Bakker J. (2015). Starling curves and central venous pressure. Crit. Care.

[54] Kenny J.-E.S. (2022). Assessing Fluid Intolerance with Doppler Ultrasonography: A Physiological Framework. Med. Sci..

[55] Kenny J.-E., Prager R., Rola P., Haycock K., Basmaji J., Hernández G. (2023). Unifying Fluid Responsiveness and Tolerance with Physiology: A dynamic interpretation of the Diamond-Forrester classification. Crit. Care Explor..

[56] Monnet X., Shi R., Teboul J.-L. (2022). Prediction of fluid responsiveness. What’s new?. Ann. Intensive Care.

[57] Abbasi A., Azab N., Nayeemuddin M., Schick A., Lopardo T., Phillips G.S., Merchant R.C., Levy M.M., Blaivas M., Corl K.A. (2020). Change in Carotid Blood Flow and Carotid Corrected Flow Time Assessed by Novice Sonologists Fails to Determine Fluid Responsiveness in Spontaneously Breathing Intensive Care Unit Patients. Ultrasound Med. Biol..

[58] Bussmann B.M., Sharma S., Mcgregor D., Hulme W., Harris T. (2019). Observational study in healthy volunteers to define interobserver reliability of ultrasound haemodynamic monitoring techniques performed by trainee doctors. Eur. J. Emerg. Med..

[59] Preau S., Bortolotti P., Colling D., Dewavrin F., Colas V., Voisin B., Onimus T., Drumez E., Durocher A., Redheuil A. (2017). Diagnostic Accuracy of the Inferior Vena Cava Collapsibility to Predict Fluid Responsiveness in Spontaneously Breathing Patients With Sepsis and Acute Circulatory Failure. Crit. Care Med..

[60] Corl K.A., Azab N., Nayeemuddin M., Schick A., Lopardo T., Zeba F., Phillips G., Baird G., Merchant R.C., Levy M.M. (2020). Performance of a 25% Inferior Vena Cava Collapsibility in Detecting Fluid Responsiveness When Assessed by Novice Versus Expert Physician Sonologists. J. Intensive Care Med..

